# “Neuroendocrine adenoma of the middle ear with the history of otitis media and carcinoma of the cheek: a case report”

**DOI:** 10.1186/1756-0500-7-532

**Published:** 2014-08-14

**Authors:** Khabti Almuhanna

**Affiliations:** Department of Otolyringoscopy, Prince Sultan Military Medical City Hospital, Riyadh, Saudi Arabia

**Keywords:** Neuroendocrine adenoma, Middle ear, Otitis media, Saudi Arabia

## Abstract

**Background:**

Neuroendocrine adenomas of the middle ear are rare benign tumors deriving from middle ear mucosal cell with both neuroendocrine and epithelial properties. Approximately one hundred cases have been reported in the literature. Here we report a patient with neuroendocrine adenoma of the middle ear with the history of otitis media; the patient earlier had received radiotherapy for the treatment of basal cell carcinoma.

**Case presentation:**

A 49- year- old Saudi man presented with a progressive hearing loss and fullness in the left ear with the history of otitis media for which he had undergone myringotomy and ventilating tubes insertion. Earlier at the age of 45 years this patient was given radiotherapy for the treatment of basal cell carcinoma in his cheek. The otoscopy showed a protruded external ear mass obscuring the tympanic membrane. Microscopy and histological examination suggested an endocrine adenoma of the middle ear. The computerized tomography scan of the temporal bone showed an extensive soft tissue mass without any osteolysis. Histological and immunohistochemical examination following surgical excision confirmed the diagnosis of neuroendocrine adenoma of the middle ear.

**Conclusion:**

A rare case of neuroendocrine adenoma of the middle ear with earlier history of otitis media and carcinoma of the cheek is presented here. Surgical excision of mass resulted in uneventful recovery. Although the etiologic factors leading to the disease is far from clear, the role of radiotherapy given for the treatment of basal cell carcinoma may not be ruled out.

## Background

Benign glandular neoplasms arising in the middle ear cavity are quite rare. Only around one hundred cases have been reported in the literature since they were first described by Derlacki [1] in 1976. It is believed that different names given to these lesions including cerminoma, ceruminous adenoma, monomorphic adenoma, adenocarcinoma and carcinoid tumor [2] represents the same tumor with the different degree of neuroendocrine differentiation, hence these lesions are now unified [3] under the name “neuroendocrine adenoma of the middle ear (NAME).” As the clinical presentation, otoscopic appearance and radiological findings of NAME are non-specific [4–7] a definitive diagnosis is based on histological and immunohistochemical findings [5].

Here, we report a case of a 49- year- old Saudi man presented with progressive hearing loss and fullness of left ear with the history of recurrent otitis media with effusion for which had undergone myrigotomy and insertion of ventilating tube. This patient had also undergone radiotherapy for the treatment of basal cell carcinoma of cheek at the age of 45 years. To the best of our knowledge, this is the first report of such a case from Saudi Arabia.

## Case presentation

A 49-year-old Saudi man consulted for an abnormal sensation of fullness in the left ear and progressive hearing loss without discharge. The patient had a history of hearing loss in the same left ear more than 20 years ago and was diagnosed as otitis media with effusion (OME). The patient had undergone left ear myringotomy and insertion of ventilating tubes twice for the treatment of OME. Four years ago this patient also received radiotherapy for the treatment of basal cell carcinoma of the cheek.

**Figure 1:**
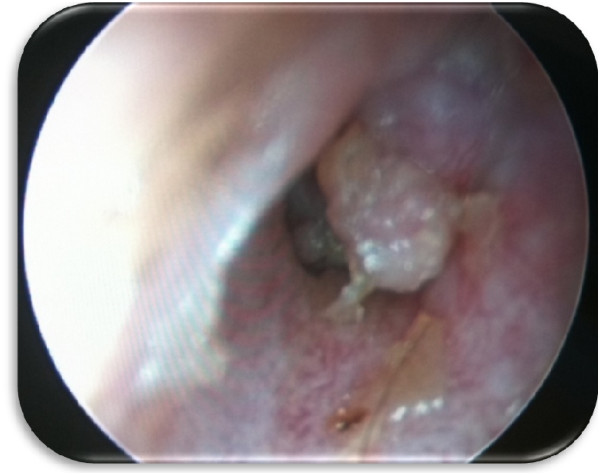
**Otoscopic view scan of left external auditory canal.** Polypoidal mass in the posterior wall of the left external auditory canal that was occluding the tympanic membrane.

**Figure 2:**
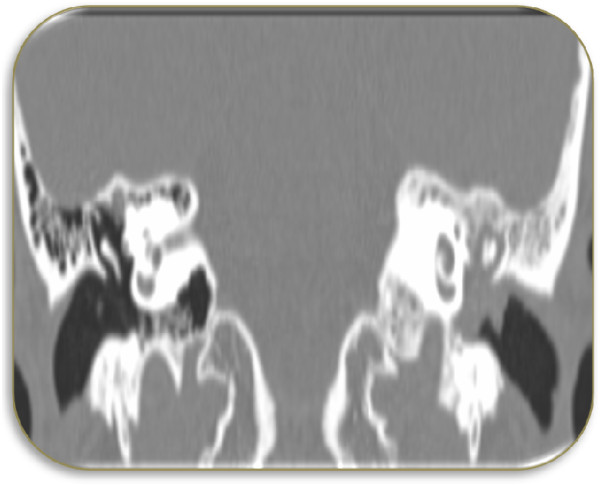
**Computed tomography scan of inner part of left external auditory canal.** An irregular soft tissue lesion seen in the inner part of the left external auditory canal just lateral to the tympanic membrane, soft tissue density is also seen implicating the Prussak’s space, epitympanum, mesotympanum and hypotympanum.

The otologic examination of left ear showed polypoidal mass in the posterior wall of the left external auditory canal that occluded the tympanic membrane (Figure 1). The facial nerve function was normal. Audiometric evaluation showed moderate conductive hearing loss. Computed tomography (CT) scan of the temporal bones (Figure 2) revealed an irregular soft tissue lesion in the inner part of the left external auditory canal just lateral to the tympanic membrane. No bony erosive changes were detected in the external auditory canal. The left mastoid air cells were opacified. A soft tissue density mass occupied the left middle ear cavity implicating the Prussak’s space, epitympanum, mesotympanum and hypotympanum. The mastoid ad antrum and mastoid antrum were also opacified by the soft tissue lesion. However, no evidence of erosive changes was found in the scutum or ossicular chain. The patient underwent excision of the left middle ear mass including the radical mastoidectomy due to extensive nature of tumor and to avoid future recurrence.

**Figure 3:**
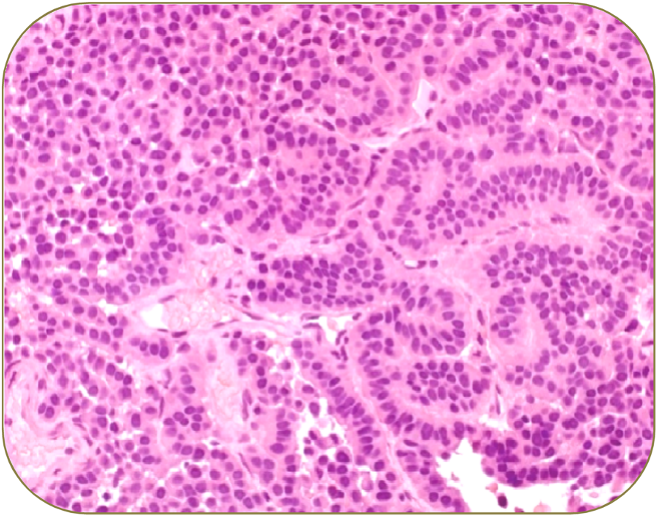
**Microscopic examination of tumor cells.** Tumor composed of glands and trabecule, uniform cuboidal cells, abundant cytoplasm, eccentrically nuclei, chromatin display a “salt-and-pepper” pattern, no mitotic activity or necrosis.

**Figure 4:**
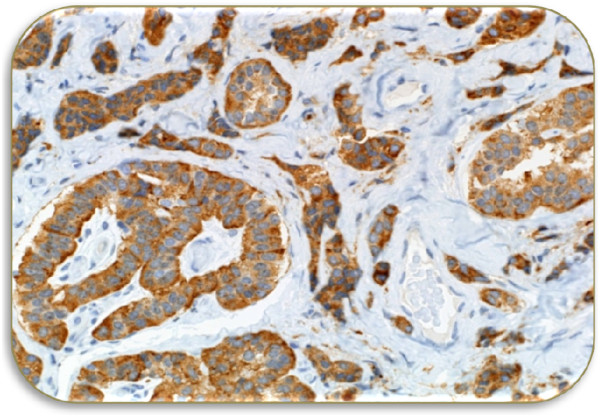
**Immunohistochemistry (positive for): Pancytokeratin CKAE l/3, Neuroendocrine markers (synaptophysin and chromogranin) and Vimentin.**

**Figure 5:**
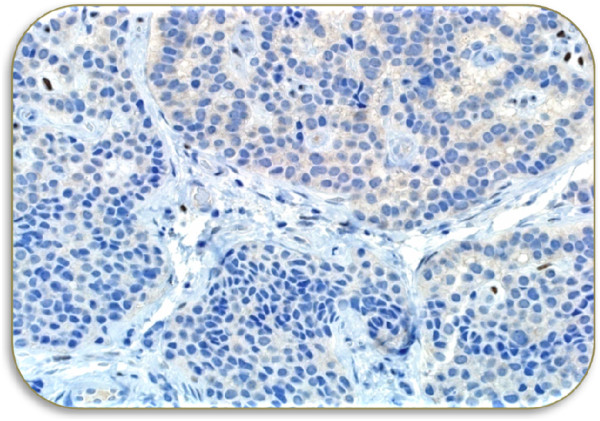
**Immunohistochemistry (negative for): S100, Desmin, CK20/CK7, TTF1 and P63.**

Microscopic examination of excised tumor revealed the presence of cuboidal and plasmacytoid cells arranged in solid sheets and in a trabecular patterns (Figure 3). Focal areas showed infiltrative pattern. The immunocytochemistry was positive for immunostains pancytokeratin (CKAE1/3), neuroendocrine markers including synaptophysin and chromogranin (Figure 4). However, they were negative for other tumor marker immunostains such as desmin, S100 Proteins, cytokeratin 20 (CK20), cytokeratin 7 (CK7), thyroid transcription factor 1 (TTF1) and tumor protein (P63) without any atypical cells (Figure 5). Based on histological and immunohistochemical findings diagnosis of neuroendocrine adenoma of the middle ear was confirmed. The post-operative period of 10 months was uneventful with no sign of recurrence.

## Discussion

Neuroendocrine adenomas of the middle ear are rare glandular lesion which account for less than 2% of all ear tumors. The mean age of NAME cases is around 45 years with no sex predominance [2]. Due to the slow growth rate and rarity of these tumors, the diagnosis of these tumors is often delayed [4]. The clinical signs, otoscopic examination and radiological findings of NAME are quite unspecific, hence the final diagnosis is confirmed by histological and immunohistochemical findings.

The patient described in this case study complained a sense of fullness and progressive hearing loss in left ear over several years. The otologic examination showed the presence of polypoidal mass in the posterior wall of external auditory canal which protruded and occluded tympanic membrane. Our case is similar to several reported cases who also showed various degree of hearing loss in NAME patients [8, 9], however majority of cases of NAME had symptoms of otalgia, otorrhea, dizziness and tinnitus [1, 4, 5, 8–10], whereas some of the reported cases were totally asymptomatic [1, 11]. Moreover most of the reported cases had intact tympanic membrane displaced medically by a retro tympanic mass [1, 8, 11]. Unlike our case facial paralysis due to compression of nerve (without invasion of lesion) was reported in several earlier cases [5]. The audiometric evaluation of our patient showed a moderate conductive hearing loss. Imaging of our patient showed irregular soft tissue lesion in the inner part of left auditory canal just lateral to the tympanic membrane without any bony erosive change. Earlier radiological studies on NAME some patients have showed homogenous, hypodense lesions of temporal bone well limited to the middle ears [8, 12]. The patient in this study underwent a complete excision of the left middle ear tumor along with radical mastoidectomy to avoid future recurrence. The microscopic examination revealed the presence of cuboidal and plasmacytoid cells arranged in a trabecular patterns. The immunohistochemistry was positive for neuroendocrine markers pancytokeratin, synaptophysin, chromogranin and vimentin immunostains confirming the presence of NAME.

Surgical excision is the only curative treatment of NAME [5, 8, 9, 11]. Generally, the treatment consists of complete surgical excision of tumor along with removal of the ossicular chain, if involved [5, 8]. Surgery should be tailored on the basis of the clinical and radiological findings; however a generalization of treatment is not advisable. A transcanal tympanotomy is performed if the lesion is small and confined to the middle ear cleft [11]. On the other hand, if the lesion fills most of the middle ear, a facial recess approach mastoidectomy is recommended [9, 11]. Torske *et al*. [5] reported a recurrence rate of 18% in their series and underlined the fact that the ossicular chain was left intact in all their patients [8]. In view of these findings a complete surgical removal of the neoplasm including encased ossicles, should be the treatment of choice. Moreover, surgical reconstruction of the tympanic membrane is simultaneously performed during the operation. However, in our case due to extensive disease we left it without reconstruction. Although the recurrence is rare in cases with initial complete excision, the patient must be followed closely for possible recurrence of the disease. No adjuvant treatment was given along with surgical excision as suggested in earlier reports [8, 9]. In our case so far a follow up of 10 months has shown no sign of recurrence. The average reported interval for the recurrence is 158 months [13]. However the reports about metastases are extremely rare and even controversial [4, 5, 7, 13]. A regular long term patient follow-up with otoscopy and audiometry is recommended. Some authors suggest systematically CT or magnetic resonance imaging (MRI) examination in order to detect recurrences [10].

The case present in this report is different from the earlier reported cases due the fact that this patient had a history of otitis media with effusion for which he had twice undergone myringotomy. Moreover, four years before the diagnosis of NAME this patient had received radiotherapy for the treatment of basal cell carcinoma of cheek. The risk of cancer from medical radiation has been reported earlier [14, 15]. Hence, a possible role of radiation in the genesis of NAME may not be ruled out.

## Conclusion

Over all middle ears adenoma with neuroendocrine differentiation is uncommon tumor. Surgical excision of the tumor is the only curative treatment. The pathogenesis of these tumors is far from clear. Earlier reports suggest that irradiation enhance the risk of tumors. In our case the patient had received radiotherapy for the treatment of carcinoma four years prior to the diagnosis of NAME. Hence, the role of radiotherapy in the etiology of NAME in this case may not be ruled out.

## Consent

“Written informed consent was obtained from the patient for publication of this case report including the images. A copy of the written consent is available for review by the Editor-in-Chief of this journal”.

## Abbreviations

NAME: Neuroendocrine adenoma of middle ear
OME: Otitis media effusion
CT: Computed tomography
MRI: Magnetic resonance imaging.

## References

[1] Derlacki E, Barney P (1976). Adenomatous tumors of the middle ear and mastoid. Laryngoscope.

[2] Berns S, Pearl G (2006). Middle ear adenoma. Arch Pathol Lab Med.

[3] Lott Limbach AA, Hoschar AP, Thompson LD, Stelow EB, Chute DJ (2012). Middle ear adenomas stain for two cell populations and lack myoepithelial cell differentiation. Head and Neck Pathol.

[4] Amble FR, Harner SG, Weiland LH, McDonald TJ, Facer GW (1993). Middle ear adenoma and adenocarcinoma. Otolaryngol Head Neck Surg.

[5] Torske K, Thompson L (2002). Adenoma versus carcinoid tumor of the middle ear: a study of 48 cases and review of the literature. Mod Pathol.

[6] Bakhos D, Lescanne E, Fetissof F, Robier A, Morinière S (2007). Neuroendocrine adenoma of the middle ear: a case study. Eur Arch Otorhinolaryngol.

[7] Saliba I, Evrard AS (2009). Middle ear glandular neoplasm: adenoma, carcinoma or adenoma with neuroendocrine differentiation: a case series. Cases J.

[8] Ayache S, Braccini F, Fernandes M, Thomassin JM (2002). Adenoma of the middle ear: a rare and misleading lesion. Otol neurol.

[9] Jones SEM, Yung MW, Orell JM, Norris A (2001). **Adenoma in the middle ear: a repor**t **of two cases**. J Laryngol Otol.

[10] Orendorz-Fraczkowska K, Jaworska M, Gawron W, Badowski R (2005). Middle ear ceruminous adenoma as a rare cause of hearing loss and vertigo: case reports. Auris Nausus larynx.

[11] Jahrsdoerfer RA, Fechner RE, Moon CN, Selman JW, Poxell JB (1983). Adenoma of the middle ear. Laryngoscope.

[12] Maintz D, Stupp C, Krueger K, Wustrow J, Lackner K (2001). MRI and CT Of adenomatous tumors of the middle ear. Neuroradiology.

[13] Ramsey MJ, Nadol JB, Pilch BZ, McKenna MJ (2005). Carcinoid tumor of the middle ear: clinical features, recurrences, and metastases. Laryngoscope.

[14] Ron E (2003). Cancer risks from medical radiation. Health Phys.

[15] Lichter MD, Karagas MR, Mott LA, Spencer SK, Stukel TA, Greenberg ER (2000). Therapeutic ionizing radiation and the incidence of basal cell carcinoma and squamous cell carcinoma. The New Hampshire Skin Cancer Study Group. Arch Dermatol.

